# A compendium of molecules involved in vector-pathogen interactions pertaining to malaria

**DOI:** 10.1186/1475-2875-12-216

**Published:** 2013-06-26

**Authors:** Sreelakshmi K Sreenivasamurthy, Gourav Dey, Manjula Ramu, Manish Kumar, Manoj K Gupta, Ajeet K Mohanty, HC Harsha, Pushkar Sharma, Nirbhay Kumar, Akhilesh Pandey, Ashwani Kumar, TS Keshava Prasad

**Affiliations:** 1 Institute of Bioinformatics, International Technology Park, Bangalore 560066, India; 2 National Institute of Malaria Research, Field Station, Goa 403001, India; 3 National Institute of Immunology, New Delhi 110067, India; 4 Department of Tropical Medicine, Tulane University School of Public Health and Tropical Medicine, New Orleans, LA 70112, USA; 5 McKusick-Nathans Institute of Genetic Medicine and Departments of Biological Chemistry, Pathology and Oncology, Johns Hopkins University School of Medicine, Baltimore, MD 21205, USA

**Keywords:** Knockdown, RNAi, Gene silencing, *Plasmodium*, *Anopheles*, Oocyst, Sporozoite

## Abstract

Malaria is a vector-borne disease causing extensive morbidity, debility and mortality. Development of resistance to drugs among parasites and to conventional insecticides among vector-mosquitoes necessitates innovative measures to combat this disease. Identification of molecules involved in the maintenance of complex developmental cycles of the parasites within the vector and the host can provide attractive targets to intervene in the disease transmission. In the last decade, several efforts have been made in identifying such molecules involved in mosquito-parasite interactions and, subsequently, validating their role in the development of parasites within the vector. In this study, a list of mosquito proteins, which facilitate or inhibit the development of malaria parasites in the midgut, haemolymph and salivary glands of mosquitoes, is compiled. A total of 94 molecules have been reported and validated for their role in the development of malaria parasites inside the vector. This compendium of molecules will serve as a centralized resource to biomedical researchers investigating vector-pathogen interactions and malaria transmission.

## Background

Malaria continues to be one of the most debilitating mosquito-borne diseases. According to the WHO World Malaria Report in 2011, 216 million cases of malaria were reported in the year 2010, resulting in 655,000 deaths, of which 86% were children below five years of age. A total of 41 different species of *Anopheles* mosquitoes act as vectors for transmission of human malaria [1]. Around 19 of them have been found to play a major role in disease-transmission across Asia-Pacific [2]. Increasing instances of pathogens gaining resistance to the first-line of drugs and replacement anti-malarials have complicated the scenario of disease control in endemic areas. One of the approaches adopted towards limiting the spread of disease is to control the vector population by the reduction of breeding sites and use of insecticides and by minimizing mosquito-human contacts through the use of mosquito nets impregnated with insecticides. Though these measures have been largely effective, the development of resistance to insecticides coupled with the increased adaptability of the pathogen susceptible strains of vectors have emphasized the need for the development of alternative approaches to intervene the transmission of malaria [3].

**Figure 1:**
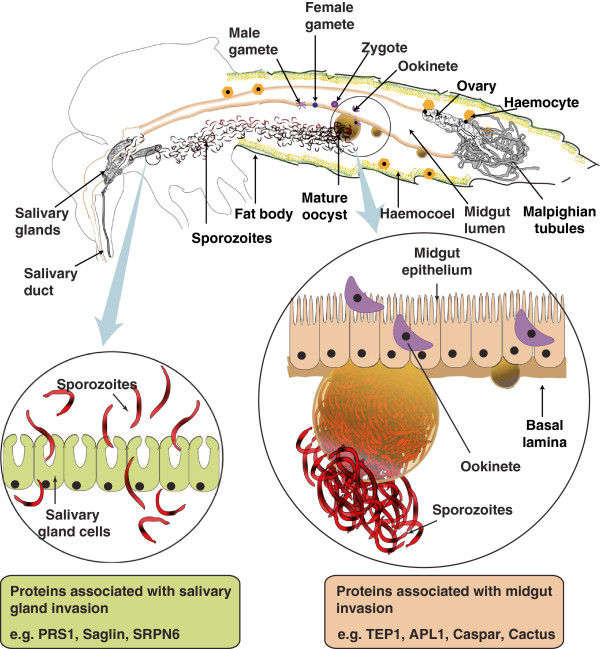
***Plasmodium *life-cycle within the mosquito.** Ingestion of *Plasmodium* infected blood by the mosquitoes is followed by reductional division in male and female gametocytes resulting in formation and fusion of male and female gametes within mosquito midgut, to form a zygote. The zygote then develops into an ookinete. The ookinete invades midgut epithelia to reach basal lamina, where it matures into oocyst. The oocysts upon maturation, release hundreds of sporozoites into haemocoel. Sporozoites migrate from midgut to salivary gland through haemolymph and then invade salivary glands. Once inside the salivary gland the sporozoites undergo further modifications and are injected into the host blood stream during a blood meal.

In their complex life cycle, during sporogonic development malaria parasites are in intimate contact with midgut, haemolymph and salivary glands of vector mosquito. The parasite, on its entry through the blood meal has to traverse through the midgut of mosquito, which acts as the first physical barrier inside the vector. Invasion of the midgut epithelia by the ookinete evokes responses within the vector to counter the assault. This constitutes the initial checkpoint for successful establishment of pathogen within the vector. The ookinetes then form oocysts, which mature in the midgut basal lamina for the next 10–12 days. Development of sporozoites heavily relies upon the mosquito metabolism. Upon maturation, each oocyst ruptures to release thousands of sporozoites into the haemocoel (Figure 1). Here, the sporozoites encounter the second challenge by the host haemocytes. The surviving sporozoites then travel to the salivary glands and gain entry into the salivary glands to undergo further modifications that are necessary to infect the subsequent mammalian host.

**Figure 2:**
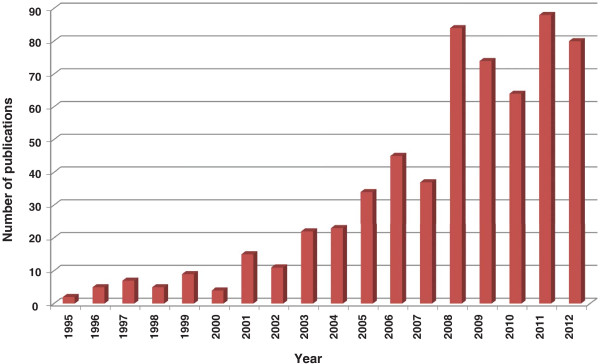
**Trend of knockdown studies in mosquitoes.** There has been an increasing trend towards knockdown studies carried out in mosquitoes recently. The graph shows a 10-fold increase in the use of gene silencing approaches to validate the role of proteins in vector-pathogen interactions.

It is known that specific proteins mediate the interactions of the *Plasmodium* parasites with mosquito tissues and regulate their maturation within their host vectors [4-6]. For example, epithelial serine protease (ESP) and cactus proteins in the midgut are known to promote the entry of *Plasmodium* ookinetes into the midgut lamina, while thioester-containing protein 1 (TEP1) and serine protease inhibitor 6 (SRPN6) proteins inhibit this process [7-12]. Similarly, the binding and invasion of salivary glands by *Plasmodium* sporozoites is reported to be mediated by the interaction of a set of surface proteins. Saglin, a receptor on salivary gland, which binds to thrombospondin-related anonymous protein on sporozoites, is one such protein involved in the internalization of sporozoites into the salivary glands of mosquito-vectors [13,14]. The difference in the vector competence of the *Anopheles* species may in part be determined by the expression of such proteins involved in the vector-pathogen interactions. RNA interference based gene silencing techniques have been successfully used in determining the role of several such mosquito proteins in the invasion of vector by malaria parasite. There has been a considerable increase in the use of this technique in the recent years (Figure 2) and many mosquito proteins, which have a role in growth and survival of malaria parasites inside the vector, have been reported. Therefore, a literature survey was performed to gather and catalog mosquito proteins with an established role in parasite development. Such a resource will be helpful in future investigations on candidate targets for strategies aimed at preventing malaria transmission.

## Methods

Scientific literature was searched for molecules involved in infection of the vector by *Plasmodium* species. In addition to a keyword search for knockdown studies in mosquitoes, several author specific searches were also performed to retrieve articles relevant to vector-pathogen interactions. These were then screened for articles reporting the effect of RNA silencing on the rate of infection. Articles with the reports of RT-PCR experiments, which had further verified the reduction of mRNA expression, were also considered for annotation. From these articles, proteins that resulted in significant changes in the rate of infection upon gene silencing were annotated. These proteins were further segregated into three major groups – i) molecules involved in midgut invasion; ii) molecules involved in melanization; and iii) molecules involved in salivary gland invasion. Molecular conditions brought about by gene silencing events, which resulted in increased numbers of oocysts at midgut lamina and sporozoites at salivary glands, were inferred to promote parasite growth and the corresponding gene or genes were deemed to be inhibitory to parasite growth. Similarly, increased melanization of ookinetes was referred to as negative effect on parasite growth.

### Summary of molecules involved in vector-*Plasmodium* interactions

**Figure 3:**
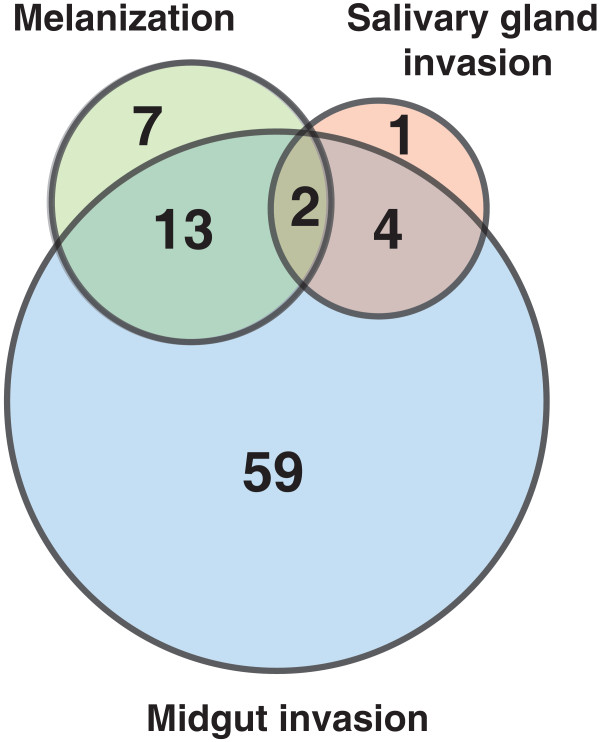
**Venn diagram representation of molecules involved in mosquito-*Plasmodium *interactions at various stages.** A total of 78 molecules were validated for their role in the midgut invasion, 22 in melanization and 7 in salivary gland invasion. Among these, SRPN6 and TEP1 were found to be involved in midgut and salivary gland invasion along with melanization of ookinetes. 13 proteins were found to be involved both in the midgut invasion and melanization and 4 molecules were involved in both midgut and salivary gland invasion.

**Figure 4:**
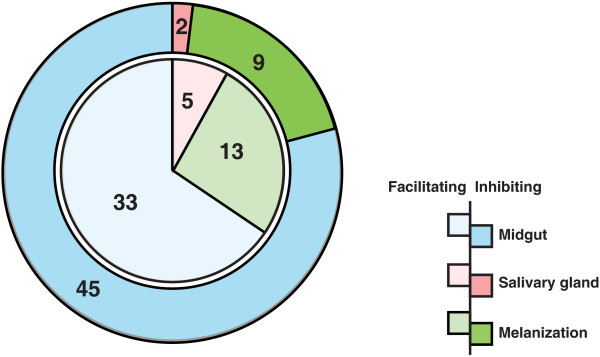
**Pie-chart representation of molecules that promote or inhibit the *Plasmodium *transmission and/or development in the vector mosquito.** The inner-pie in a lighter shade represents the number of molecules that are facilitating the invasion or transmission of the *Plasmodium* in mosquito, while the darker outer-pie represents the molecules antagonists to *Plasmodium* development in mosquito.

This article presents a resource of 94 evidence-based molecules involved in vector-pathogen interactions and vector immune responses. From an initial list of 572 articles retrieved using different search strategies, a subset of 55 articles contained reports of vector-pathogen interactions, from which the information was compiled into this compendium. Proteins, either promoting or inhibiting parasite growth were catalogued with associated information on the number of oocysts produced, rate of melanization of ookinetes in midgut and/or number of sporozoites found in the salivary glands upon different experimental conditions (Figure 3). The most common method employed to determine the role of mosquito protein infection was to knockdown the gene expression and then measure its effect in terms of oocyst count. Among 78 proteins identified by this strategy, oocyst count decreased on silencing 33 genes and increased in case of 45 genes (Figure 4). In other words, former set of proteins were found to promote midgut invasion whereas the latter to result in inhibition. Only six studies reported investigation of proteins, which are involved in salivary gland invasion (Figure 4). At least 22 molecules were found to affect the melanization process upon silencing. Knocking down of 13 and 9 genes resulted in increased and decreased melanization, respectively (Figure 4). Some of these molecules had no effect on either the oocyst or sporozoite counts, when silenced individually. However, they showed significant variation when silenced in combination with other genes. The role of certain proteins, including gastrin/cholecystokinin receptor 1 (GPRCCK1) and leucine-rich repeat immune protein (LRIM1), was dependent on the species of mosquitoes in which the experiment was performed and the *Plasmodium* species used for infecting them [15-17].

### Molecules involved in midgut invasion

Fusion of male and female gametes within the midgut of the mosquitoes results in the formation of zygotes and ookinetes, which then invade epithelium of the midgut and migrate to the basal lamina to form oocysts. The invasion of mosquito midgut by pathogens provides a critical step that involves adhesion to the midgut epithelia of the vector. Successful recognition and invasion of midgut epithelia by ookinetes is dependent on molecules that either facilitate or inhibit the parasite development. For instance, alanyl aminopeptidase N (APN1) was reported as a surface recognition molecule in the mosquito midgut [18]. Epithelial invasion by the ookinetes is followed by the transformation of ookinetes to oocysts in the basal lamina. Seventy eight mosquito proteins were shown to be involved in midgut invasion by ookinetes and parasite survival, by survey of scientific literature.

Proteins such as TEP1 and LRIM1 are known to recognize the invading ookinetes and trigger immune responses in mosquitoes (Additional file 1) [8,16,19,20]. C-type lectin 4 (CTL4), caspar and cactus are found to negatively regulate the immune responses, resulting in a decreased oocyst count upon their silencing [8,21]. The proteins which generated complete refractoriness in the vector upon silencing may be the target of choice for future studies to block disease transmission. Heat shock protein, HSC3 resulted in decreased oocyst count upon knocking down in case of *Anopheles gambiae* and *Plasmodium falciparum*. However, in case of *Anopheles stephensi* infected with *Plasmodium yoelii*, it resulted in increased oocyst count [16]. Similarly gastrin/cholecystokinin receptor 1 (GPRCCK1) also showed increase in the oocyst count upon infection of *An. gambiae* with *Plasmodium berghei* and decrease upon infection with *P. falciparum* (Additional file 2) [15].

Certain proteins such as clip-domain serine protease 17 (CLIPB17) showed no significant change in the number of oocysts upon its silencing. However, in combination with CTL4*,* silencing of CLIPB17 resulted in a significant decrease of oocysts (Additional files 1 and 3) [21]. Similarly, ARC P21 and ARC P41 subunits of the actin related protein complex shows no effect on the number of oocysts formed, when silenced individually. However, a double knockdown of these genes resulted in the formation of larger number of oocysts [22].

### Molecules involved in melanization

Of the many ookinetes that are formed in the mosquito midgut, only a handful develops into a mature oocyst that upon rupturing, release sporozoites into the haemocoel. Sporozoites then migrate towards salivary glands of mosquito through haemolymph. Phagocytosis and melanin deposition on the surface of invading parasites represents the major immune responses in arthropods [23,24]. The mechanism of these responses is not completely understood. However, various models have been proposed. Melanization of the invading ookinetes possibly results in the reduced formation of oocysts, thereby preventing transmission.

A total of 22 molecules have been identified thus far, to play a role in the melanization of ookinetes within the mosquitoes. Gene silencing studies undertaken to understand the biology of melanogenesis, revealed the role of serine proteases, such as CLIPB4, CLIPB8 and TEP1 [10,21]. Silencing of these serine proteases resulted in the reduction in melanization, when infected with *P. berghei.* Among the 22 genes affecting the rate of melanization, two proteins SRPN6 and TEP1 were found to affect oocyst and sporozoite counts along with melanization of ookinetes. SRPN6 was reported to increase the melanization rates in refractory strains of *An. gambiae*, but not in susceptible G3 strain or *An. stephensi*. However, knockdown of SRPN6 in combination with CTL4 was reported to significantly increase the rate of melanization in susceptible G3 strain of *An. gambiae*[12]. Fourteen of them were found to be associated with the oocyst invasion and melanization. Silencing of seven genes including clip-domain serine proteases, such as CLIPA2 and CLIPA5 were found to increase the rate of melanization and decrease the oocyst count [17,21,25,26]. Knocking down of 3 genes (*APL1C*, *LRIM1* and *TEP1*) showed decreased melanization of ookinetes and increased number of oocysts [8,10,16,17,20,27-34]. However, silencing of CLIPB3 and CLIPA8 resulted in the reduction of both melanization and oocyst counts (Additional files 1 and 4, Figure 3) [21]. Some investigations revealed that melanization may not necessarily affect parasite invasion, and may be involved in the disposal of dead parasites [21,35].

### Molecules involved in salivary gland invasion

Sporozoites that survive mosquito immune response in the midgut lamina and haemocoel of mosquitoes migrate to salivary glands through haemolymph. Losses of up to 80 - 90% sporozoites have been reported, during their migration to the salivary glands through haemolymph by various mechanisms that are not yet completely understood [36]. Effective and specific associations of sporozoite surface antigens such as TRAP, with receptors such as saglin on the salivary glands of mosquitoes initiate the salivary gland invasion by sporozoites [13]. *Plasmodium* responsive salivary 1 (PRS1), ESP, peptide-O-xylosyltransferase 1 (OXT1) and SRPN6 have been identified to play a crucial role in parasite invasion of both midgut and salivary glands. Silencing of PRS1, ESP*,* retinoid and fatty-acid binding glycoprotein (RFABG) and OXT1 resulted in decreased oocyst and sporozoite counts (Additional files 1 and 5, Figure 3), whereas SRPN6 and TEP1 silencing resulted in an increase in production of oocysts and sporozoites [7,37-40].

The sporozoites undergo changes in their membrane proteins upon salivary gland invasion, which enable them to successfully infect the vertebral hosts [41]. Salivary gland invasion by the sporozoites and their development within is an essential step in the parasite life cycle and effective transmission of *Plasmodium*. Blocking of these processes could disrupt the parasite development in mosquitoes and suppress malaria transmission.

## Conclusions

Blocking the vector-parasite interactions can be one of the novel approaches to control transmission of vector borne infections. Several proteins of mosquitoes have been involved in the regulation of maturation of malaria parasites within the vector. Transmission-blocking vaccine (TBV) is one of the strategies, which specifically target such proteins and block parasite invasion and survival. TBVs when injected in vertebrate host as purified antigens induce production of antibodies in the host against the corresponding antigens. These antibodies then interfere with the parasite transmission in the vector following blood meal on an infected and vaccinated individual [42]. Furthermore, engineered anti-*Plasmodium* immunity in transgenic mosquitoes has shown increased resistance in the vector against the pathogen [43]. Such genetically modified insects have been successful in replacing the wild type population [44].

Among the molecules listed in this study, 40 proteins were found to assist in parasite survival at different stages. Proteins which are involved in vector immunity resulted in increased number of oocysts or sporozoites upon being knocked down, showing their involvement in suppressing the parasite during infection. A resource of such proteins would provide a platform for other studies, to identify the potential targets and to translate the outcome as possible mechanism for malaria control. Such novel approaches along with the conventional ways of vector control and infection treatment may lead to accomplish the vision of malaria eradication.

## Abbreviations

WHO: World Health Organization; TBV: Transmission-blocking vaccine.

## Competing interests

The authors declare that they have no competing interests.

## Authors’ contributions

TSKP, AP, AK, HCH and NK conceived and planned the study. SKS, GD, MR, MK, MKG and AKM curated the data. SKS and GD compiled the data. SKS, HCH and TSKP wrote the manuscript. GD drew all diagrams. SKS prepared all tables. PS, NK, AK and AP provided critical inputs and revised the manuscript. All authors read and approved the final manuscript.

## Supplementary Material

Additional file 1 **Molecules affecting oocyst counts.** The file includes the molecules that aid or inhibit the oocyst formation in mosquito midgut. The mosquito proteins that aid oocyst formation are known to be agonistic and those that inhibit the oocyst formation are known to be antagonistic in nature. Click here for file

Additional file 2 **Molecules that differentially affect the *Plasmodium *development depending on the mosquito and *Plasmodium *species.** The file includes the list of molecules that have different effects on the malarial parasite in different mosquito and/or *plasmodium* species. Depending on the vector and parasite species, these molecules either aid or inhibit oocyst formation. Click here for file

Additional file 3 **Molecules affecting *Plasmodium *development studied by multiple knockdowns.** The file consists of molecules, which have no or negligible effect upon silencing individually. However, when these molecules are silenced in combination with some other molecules, they affect the parasitic growth either positively or negatively. Click here for file

Additional file 4 **Molecules affecting melanization of ookinetes.** The file lists the molecules, which have been shown to be involved in the process of inhibiting or promoting the parasitic growth through ookinete melanization. Click here for file

Additional file 5 **Molecules affecting sporozoite counts.** The file consists of molecules that promote or inhibit the development of sporozoites. The molecules that increase the sporozoite count are agonists and those that reduce sporozoite counts are known as antagonists. Click here for file
