# Supplementary material for: A compendium of molecules involved in vector-pathogen interactions pertaining to malaria
Source: Malar J. 2013 Jun 26;12:216. doi: 10.1186/1475-2875-12-216 (PMC3734095; doi:10.1186/1475-2875-12-216)
Supplement: Additional file 1 — Molecules affecting oocyst counts. The file includes the molecules that aid or inhibit the oocyst formation in mosquito midgut. The mosquito proteins that aid oocyst formation are known to be agonistic and those that inhibit the oocyst formation are known to be antagonistic in nature. [file 1475-2875-12-216-S1.docx]

**Additional Table 1**: **Molecules affecting oocyst counts**

The table includes the molecules that aid or inhibit the oocyst formation in mosquito midgut. The mosquito proteins that aid oocyst formation are known to be agonistic and those that inhibit the oocyst formation are known to be antagonistic in nature.

| ***Agonistic molecules:* those that aid *Plasmodium* development in mosquito**  **(oocyst number decreases upon knock-down)** | | | | | |
| --- | --- | --- | --- | --- | --- |
|  | **Protein** | | **Protein name** | **VectorBase ID** | **References** |
| 1 | ANT | | Adenine nucleotide translocator | AGAP006782 | *Oliveira, JH et al., 2011.* |
| 2 | ArgK | | Arginine kinase | AGAP005627 | *Jaramillo-Gutierrez, G et al., 2009. Brandt, SM et al., 2008.* |
| 3 | CACT | | Cactus | AGAP007938 | *Garver, LS et al., 2009.*  *Zou, Z et al., 2011.* |
| 4 | Caspar | | Caspar | AGAP006473 | *Garver, LS et al., 2009.*  *Garver, LS et al., 2012.* |
| 5 | CLIPA2 | | CLIP-domain serine protease subfamily A 2 | AGAP011790 | *Volz, J et al., 2006.* |
| 6 | CLIPA5 | | CLIP-domain serine protease subfamily A 5 | AGAP011787 | *Volz, J et al., 2006.* |
| 7 | CLIPA8 | | CLIP-domain serine protease subfamily A 8 | AGAP010731 | *Volz, J et al., 2006.* |
| 8 | CLIPB3 | | CLIP-domain serine protease subfamily B 3 | AGAP003249 | *Volz, J et al., 2006.* |
| 9 | CLSP2 | | Serine protease, putative | AAEL011616 | *Shin, SW et al., 2011.* |
| 10 | CP | | F-actin capping protein | AGAP007864 | *Vlachou, D et al., 2005.* |
| 11 | CPR | | Cytochrome P450 reductase | AGAP000500 | *Félix, RC & Silveira, H, 2011.* |
| 12 | CTL4 | | C-type lectin 4 | AGAP005335 | *Volz, J et al., 2006.* |
| 13 | CTLMA2 | | CTL mannose binding 2 | AGAP005334 | *Cohuet, A et al., 2006.* |
| 14 | DUOX | | Dual oxidase | AGAP009978 | *Kumar, S et al., 2010.* |
| 15 | ESP | | *Anopheles gambiae* epithelial serine protease | AGAP010240 | *Rodrigues, J et al., 2012.* |
| 16 | Gelsolin | | Gelsolin | AGAP011369 | *Vlachou, D et al., 2005.* |
| 17 | GPRNPY3 | | Putative neuropeptide Y receptor | AGAP012378 | *Mendes, AM et al., 2011.* |
| 18 | GSTT1 | | Glutathione-S-transferase theta-1 | AGAP000761 | *Jaramillo-Gutierrez, G et al., 2009.* |
| 19 | IMPer | | Immunomodulatory peroxidase | AGAP013327 | *Kumar, S et al., 2010.* |
| 20 | LANB2 | | Laminin gamma 1 | AGAP007629 | *Arrighi, RB et al., 2005.* |
| 21 | LYSC-1 | | Lysozyme c-1 | AGAP007347 | *Lapcharoen, P et al., 2012.*  *Kajla, MK et al., 2011.* |
| 22 | OXR1 | | Oxidation Resistance gene 1 | AGAP001751 | *Jaramillo-Gutierrez, G et al., 2010. Brandt, SM et al., 2008.* |
| 23 | OXT1 | | Peptide-O-xylosyltransferase 1 | AGAP005811 | *Dinglasan, RR et al., 2007.* |
| 24 | PIAS | | Protein Inhibitor of Activated STAT | AGAP005031 | *Zou, Z et al., 2011.* |
| 25 | PRS1 | | *Plasmodium* responsive salivary 1 | AGAP006102 | *Chertemps, T et al., 2010.* |
| 26 | RFABG | | Retinoid and fatty-acid binding glycoprotein, also known as lipophorin or ApoII/I | AGAP001826 | *Mendes, AM et al., 2008.*  *Vlachou, D et al., 2005. Rono, MK et al., 2010. Cheon, HM et al., 2006.* |
| 27 | SCRBQ2 | | Croquemort ortholog | AGAP010133 | *González-Lázaro, M et al., 2009.* |
| 28 | SDR1 | | Short-chain dehydrogenases/reductases | AGAP002521 | *Berois, M et al., 2012.* |
| 29 | SRPN2 | | Serine protease inhibitor 2 (also known as serpin 2) | AGAP006911 | *Michel, K et al., 2005.*  *Michel, K et al., 2006.* |
| 30 | Vg | | Vitellogenin | AGAP004203 | *Rono, MK et al., 2010.* |
| 31 | AgaP_ AGAP004016 | | - | AGAP004016 | *Pinto, SB et al., 2009.* |
| 32 | AgaP_ AGAP006914 | | - | AGAP006914 | *Pinto, SB et al., 2009.* |
| 33 | AgaP_ AGAP013527 | | - | AGAP013527 | *Pinto, SB et al., 2009.* |
| ***Antagonistic molecules:* those that prevent *Plasmodium* development in mosquito**  **(oocyst number increases upon knock-down)** | | | | | |
|  | | **Protein** | **Protein name** | **VectorBase ID** | **References** |
| 1 | | APL1B | *Anopheles* *Plasmodium*-responsive Leucine-rich repeat protein 1B | AGAP007035 | *Garver, LS et al., 2012.* |
| 2 | | APL1C | *Anopheles* *Plasmodium*-responsive Leucine-rich repeat protein 1C | AGAP007033 | *Riehle, MM et al., 2008. Mitri, C et al., 2009.*  *Garver, LS et al., 2012.* |
| 3 | | APOD | Apo lipoprotein | AGAP002593 | *Dong, Y et al., 2006.* |
| 4 | | ApoLp-III | Apolipophorin-III | AGAP013365 | *Gupta, L et al., 2010.* |
| 5 | | CASPL1 | Caspase long class | AGAP011693 | *Garver, LS et al., 2012.* |
| 6 | | Ciboulot | Beta thymosin family | AGAP000235 | *Vlachou, D et al., 2005.* |
| 7 | | CLIP1 | CLIP-domain serine protease 1 | ENSANGT00000020158 | *Dong, Y et al., 2006.* |
| 8 | | CLIPB14 | CLIP-domain serine protease subfamily B14 | AGAP010833 | *Volz, J et al., 2005.* |
| 9 | | CLIPB15 | CLIP-domain serine protease subfamily B15 | AGAP009844 | *Volz, J et al., 2005.* |
| 10 | | FADD | Fas-Associated Death Domain | AGAP007173 | *Garver, LS et al., 2012.* |
| 11 | | FBN6 | Fibrinogen domain immunolectin 6 | AGAP011231 | *Dong, Y et al., 2009.* |
| 12 | | FBN8 | Fibrinogen domain immunolectin 8 | AGAP011223 | *Dong, Y et al., 2006. Dong, Y et al., 2009.* |
| 13 | | FBN9 | Fibrinogen domain immunolectin 9 | AGAP011197 | *Dong, Y et al., 2006. Garver, LS et al., 2009.  Dong, Y et al., 2009.* |
| 14 | | FBN39 | Fibrinogen domain immunolectin 39 | AGAP000806 | *Dong, Y et al., 2006. Dong, Y et al., 2009.* |
| 15 | | GAM1 | Gambicin | AGAP008645 | *Dong, Y et al., 2006.* |
| 16 | | GNBPA2 | Gram-negative bacteria binding protein A2 | AGAP012409 | *Warr, E et al., 2008.* |
| 17 | | GNBPB1 | Gram-negative bacteria binding protein B1 | AGAP004455 | *Dong, Y et al., 2006.* |
| 18 | | GNBPB3 | Gram-negative bacteria binding protein B3 | AGAP002799 | *Warr, E et al., 2008.* |
| 19 | | GNBPB4 | Gram-negative bacteria binding protein B4 | AGAP002796 | *Warr, E et al., 2008.* |
| 20 | | GPGRP1 | Gastrin/bombesin receptor 1 | AGAP011452 | *Mendes, AM et al., 2011.* |
| 21 | | GPGRP2 | Gastrin/bombesin receptor 2 | AGAP003631 | *Mendes, AM et al., 2011.* |
| 22 | | GSTT2 | Glutathione-S-transferase theta-2 | AGAP000888 | *Jaramillo-Gutierrez, G et al., 2009.* |
| 23 | | IKK-gamma | Inhibitor of kappa B kinase gamma | AGAP005933 | *Garver, LS et al., 2012.* |
| 24 | | IMD | Immunodeficiency domain | AGAP004959 | *Meister, S et al., 2005.*  *Garver, LS et al., 2012.* |
| 25 | | IRSP1 | Infection responsive secreted peptide 1 | AGAP006421 | *Dong, Y et al., 2006.* |
| 26 | | IRSP5 | Infection responsive secreted peptide 5 | AGAP000151 | *Dong, Y et al., 2006.* |
| 27 | | JNK | Jun N-terminal Kinase | AGAP009461 | *Jaramillo-Gutierrez, G et al., 2011.* |
| 28 | | LRIM1 | Leucine-Rich Immune Molecule 1 | AGAP006348 | *Habtewold, T et al., 2008.  Jaramillo-Gutierrez, G et al., 2009. Riehle, MM et al., 2006. Povelones, M et al., 2009. Osta, MA et al., 2004.  Cohuet, A et al., 2006.*  *Garver, LS et al., 2012.* |
| 29 | | LRIM2 | Leucine-Rich Immune Molecule 2 also known as APL2 or LRRD7 | AGAP005693 | *Habtewold, T et al., 2008. Jaramillo-Gutierrez, G et al., 2009. Riehle, MM et al., 2006. Povelones, M et al., 2009.  Dong, Y et al., 2006. Garver, LS et al., 2009.*  *Garver, LS et al., 2012.* |
| 30 | | MC1 | Mitochondrial carrier 1 | AGAP001297 | *Gonçalves, RL et al., 2012.* |
| 31 | | MDL1 | MD2-like receptor 1 | AGAP012352 | *Dong, Y et al., 2006.* |
| 32 | | PGRPLC | PGN Recognition Protein LC | AGAP005203 | *Meister, S et al., 2009.* |
| 33 | | REL2 | Relish | AGAP006747 | *Meister, S et al., 2005.*  *Garver, LS et al., 2012.* |
| 34 | | Sp SNAKE like | Serine protease SNAKE like | AGAP012946 | *Harris, C et al., 2010.* |
| 35 | | SRPN6 | Serine protease inhibitor 6 (also known as serpin 6) | AGAP009212 | *Abraham, EG et al., 2005.* |
| 36 | | STAT | Signal Transducers and Activators of Transcription | AGAP000099 | *Bahia, AC et al., 2011.* |
| 37 | | TEP1 | Thioester-containing protein 1 | AGAP010815 | *Dong, Y et al., 2006. Habtewold, T et al., 2008.  Jaramillo-Gutierrez, G et al., 2009.*  *Garver, LS et al., 2009. Dong, Y et al., 2009. Rono, MK et al., 2010.  Molina-Cruz, A et al., 2012.*  *Garver, LS et al., 2012.*  *Blandin, S et al., 2004.* |
| 38 | | Tetrasp. | Tetraspanin | AGAP005233 | *Jaramillo-Gutierrez, G et al., 2009. Brandt, SM et al., 2008.* |
| 39 | | WASP | Wiskott-Aldrich syndrome protein | AGAP001081 | *Vlachou, D et al., 2005.  Mendes, AM et al., 2008.* |
| 40 | | LL3 | LITAF-like 3 | AGAP009053 | *Smith, RC et al., 2012.* |
| 41 | | Kto | Kohtalo (also known as Med12 or TRAP230) | AGAP002523 | *Chen, Y et al., 2012.* |
| 42 | | Skd | Skuld (also known as Med13 or TRAP240) | AGAP006436 | *Chen, Y et al., 2012.* |
| 43 | | AgaP_ AGAP005761 | - | AGAP005761 | *Pinto, SB et al., 2009.* |
| 44 | | AgaP_ AGAP009642 | - | AGAP009642 | *Pinto, SB et al., 2009.* |
| 45 | | AgaP_ AGAP010325 | - | AGAP010325 | *Pinto, SB et al., 2009.* |

**References**

1. Oliveira JH, Goncalves RL, Oliveira GA, Oliveira PL, Oliveira MF, Barillas-Mury C: **Energy metabolism affects susceptibility of *Anopheles gambiae* mosquitoes to *Plasmodium* infection.** *Insect Biochem Mol Biol* 2011, **41:**349-355.

2. Jaramillo-Gutierrez G, Rodrigues J, Ndikuyeze G, Povelones M, Molina-Cruz A, Barillas-Mury C: **Mosquito immune responses and compatibility between *Plasmodium* parasites and *anopheline* mosquitoes.** *BMC Microbiol* 2009, **9:**154.

3. Brandt SM, Jaramillo-Gutierrez G, Kumar S, Barillas-Mury C, Schneider DS: **Use of a *Drosophila* model to identify genes regulating *Plasmodium* growth in the mosquito.** *Genetics* 2008, **180:**1671-1678.

4. Garver LS, Dong Y, Dimopoulos G: **Caspar controls resistance to *Plasmodium falciparum* in diverse anopheline species.** *PLoS Pathog* 2009, **5:**e1000335.

5. Zou Z, Souza-Neto J, Xi Z, Kokoza V, Shin SW, Dimopoulos G, Raikhel A: **Transcriptome analysis of *Aedes aegypti* transgenic mosquitoes with altered immunity.** *PLoS Pathog* 2011, **7:**e1002394.

6. Garver LS, Bahia AC, Das S, Souza-Neto JA, Shiao J, Dong Y, Dimopoulos G: ***Anopheles* Imd pathway factors and effectors in infection intensity-dependent anti-*Plasmodium* action.** *PLoS Pathog* 2012, **8:**e1002737.

7. Volz J, Muller HM, Zdanowicz A, Kafatos FC, Osta MA: **A genetic module regulates the melanization response of *Anopheles* to *Plasmodium*.** *Cell Microbiol* 2006, **8:**1392-1405.

8. Shin SW, Zou Z, Raikhel AS: **A new factor in the *Aedes aegypti* immune response: CLSP2 modulates melanization.** *EMBO Rep* 2011, **12:**938-943.

9. Vlachou D, Schlegelmilch T, Christophides GK, Kafatos FC: **Functional genomic analysis of midgut epithelial responses in *Anopheles* during *Plasmodium* invasion.** *Curr Biol* 2005, **15:**1185-1195.

10. Felix RC, Silveira H: **The interplay between tubulins and P450 cytochromes during *Plasmodium berghei* invasion of *Anopheles gambiae* midgut.** *PLoS ONE* 2011, **6:**e24181.

11. Cohuet A, Osta MA, Morlais I, Awono-Ambene PH, Michel K, Simard F, Christophides GK, Fontenille D, Kafatos FC: ***Anopheles* and *Plasmodium*: from laboratory models to natural systems in the field.** *EMBO Rep* 2006, **7:**1285-1289.

12. Kumar S, Molina-Cruz A, Gupta L, Rodrigues J, Barillas-Mury C: **A peroxidase/dual oxidase system modulates midgut epithelial immunity in *Anopheles gambiae*.** *Science* 2010, **327:**1644-1648.

13. Rodrigues J, Oliveira GA, Kotsyfakis M, Dixit R, Molina-Cruz A, Jochim R, Barillas-Mury C: **An epithelial serine protease, AgESP, is required for *Plasmodium* invasion in the mosquito *Anopheles gambiae*.** *PLoS ONE* 2012, **7:**e35210.

14. Mendes AM, Awono-Ambene PH, Nsango SE, Cohuet A, Fontenille D, Kafatos FC, Christophides GK, Morlais I, Vlachou D: **Infection intensity-dependent responses of *Anopheles gambiae* to the African malaria parasite *Plasmodium falciparum*.** *Infect Immun* 2011, **79:**4708-4715.

15. Arrighi RB, Lycett G, Mahairaki V, Siden-Kiamos I, Louis C: **Laminin and the malaria parasite's journey through the mosquito midgut.** *J Exp Biol* 2005, **208:**2497-2502.

16. Lapcharoen P, Komalamisra N, Rongsriyam Y, Wangsuphachart V, Dekumyoy P, Prachumsri J, Kajla MK, Paskewitz SM: **Investigations on the role of a lysozyme from the malaria vector *Anopheles dirus* during malaria parasite development.** *Dev Comp Immunol* 2012, **36:**104-111.

17. Kajla MK, Shi L, Li B, Luckhart S, Li J, Paskewitz SM: **A new role for an old antimicrobial: lysozyme c-1 can function to protect malaria parasites in *Anopheles* mosquitoes.** *PLoS ONE* 2011, **6:**e19649.

18. Jaramillo-Gutierrez G, Molina-Cruz A, Kumar S, Barillas-Mury C: **The *Anopheles* *gambiae* oxidation resistance 1 (OXR1) gene regulates expression of enzymes that detoxify reactive oxygen species.** *PLoS ONE* 2010, **5:**e11168.

19. Dinglasan RR, Kalume DE, Kanzok SM, Ghosh AK, Muratova O, Pandey A, Jacobs-Lorena M: **Disruption of *Plasmodium falciparum* development by antibodies against a conserved mosquito midgut antigen.** *Proc Natl Acad Sci U S A* 2007, **104:**13461-13466.

20. Chertemps T, Mitri C, Perrot S, Sautereau J, Jacques JC, Thiery I, Bourgouin C, Rosinski-Chupin I: ***Anopheles gambiae* PRS1 modulates *Plasmodium* development at both midgut and salivary gland steps.** *PLoS ONE* 2010, **5:**e11538.

21. Mendes AM, Schlegelmilch T, Cohuet A, Awono-Ambene P, De Iorio M, Fontenille D, Morlais I, Christophides GK, Kafatos FC, Vlachou D: **Conserved mosquito/parasite interactions affect development of *Plasmodium falciparum* in Africa.** *PLoS Pathog* 2008, **4:**e1000069.

22. Rono MK, Whitten MM, Oulad-Abdelghani M, Levashina EA, Marois E: **The major yolk protein vitellogenin interferes with the anti-*plasmodium* response in the malaria mosquito *Anopheles gambiae*.** *PLoS Biol* 2010, **8:**e1000434.

23. Cheon HM, Shin SW, Bian G, Park JH, Raikhel AS: **Regulation of lipid metabolism genes, lipid carrier protein lipophorin, and its receptor during immune challenge in the mosquito *Aedes aegypti*.** *J Biol Chem* 2006, **281:**8426-8435.

24. Gonzalez-Lazaro M, Dinglasan RR, Hernandez-Hernandez Fde L, Rodriguez MH, Laclaustra M, Jacobs-Lorena M, Flores-Romo L: ***Anopheles gambiae* Croquemort SCRBQ2, expression profile in the mosquito and its potential interaction with the malaria parasite *Plasmodium berghei*.** *Insect Biochem Mol Biol* 2009, **39:**395-402.

25. Berois M, Romero-Severson J, Severson DW: **RNAi knock-downs support roles for the mucin-like (AeIMUC1) gene and short-chain dehydrogenase/reductase (SDR) gene in *Aedes aegypti* susceptibility to *Plasmodium gallinaceum*.** *Med Vet Entomol* 2012, **26:**112-115.

26. Michel K, Budd A, Pinto S, Gibson TJ, Kafatos FC: ***Anopheles gambiae* SRPN2 facilitates midgut invasion by the malaria parasite *Plasmodium berghei*.** *EMBO Rep* 2005, **6:**891-897.

27. Michel K, Suwanchaichinda C, Morlais I, Lambrechts L, Cohuet A, Awono-Ambene PH, Simard F, Fontenille D, Kanost MR, Kafatos FC: **Increased melanizing activity in *Anopheles gambiae* does not affect development of *Plasmodium falciparum*.** *Proc Natl Acad Sci U S A* 2006, **103:**16858-16863.

28. Pinto SB, Lombardo F, Koutsos AC, Waterhouse RM, McKay K, An C, Ramakrishnan C, Kafatos FC, Michel K: **Discovery of *Plasmodium* modulators by genome-wide analysis of circulating hemocytes in *Anopheles gambiae*.** *Proc Natl Acad Sci U S A* 2009, **106:**21270-21275.

29. Riehle MM, Xu J, Lazzaro BP, Rottschaefer SM, Coulibaly B, Sacko M, Niare O, Morlais I, Traore SF, Vernick KD: ***Anopheles gambiae* APL1 is a family of variable LRR proteins required for Rel1-mediated protection from the malaria parasite, *Plasmodium berghei*.** *PLoS ONE* 2008, **3:**e3672.

30. Mitri C, Jacques JC, Thiery I, Riehle MM, Xu J, Bischoff E, Morlais I, Nsango SE, Vernick KD, Bourgouin C: **Fine pathogen discrimination within the APL1 gene family protects *Anopheles gambiae* against human and rodent malaria species.** *PLoS Pathog* 2009, **5:**e1000576.

31. Dong Y, Aguilar R, Xi Z, Warr E, Mongin E, Dimopoulos G: ***Anopheles gambiae* immune responses to human and rodent *Plasmodium* parasite species.** *PLoS Pathog* 2006, **2:**e52.

32. Gupta L, Noh JY, Jo YH, Oh SH, Kumar S, Noh MY, Lee YS, Cha SJ, Seo SJ, Kim I, et al: **Apolipophorin-III mediates antiplasmodial epithelial responses in *Anopheles gambiae* (G3) mosquitoes.** *PLoS ONE* 2010, **5:**e15410.

33. Volz J, Osta MA, Kafatos FC, Muller HM: **The roles of two clip domain serine proteases in innate immune responses of the malaria vector *Anopheles gambiae*.** *J Biol Chem* 2005, **280:**40161-40168.

34. Dong Y, Dimopoulos G: ***Anopheles* fibrinogen-related proteins provide expanded pattern recognition capacity against bacteria and malaria parasites.** *J Biol Chem* 2009, **284:**9835-9844.

35. Warr E, Das S, Dong Y, Dimopoulos G: **The Gram-negative bacteria-binding protein gene family: its role in the innate immune system of *Anopheles gambiae* and in anti-*Plasmodium* defence.** *Insect Mol Biol* 2008, **17:**39-51.

36. Habtewold T, Povelones M, Blagborough AM, Christophides GK: **Transmission blocking immunity in the malaria non-vector mosquito *Anopheles quadriannulatus* species A.** *PLoS Pathog* 2008, **4:**e1000070.

37. Riehle MM, Markianos K, Niare O, Xu J, Li J, Toure AM, Podiougou B, Oduol F, Diawara S, Diallo M, et al: **Natural malaria infection in *Anopheles gambiae* is regulated by a single genomic control region.** *Science* 2006, **312:**577-579.

38. Povelones M, Waterhouse RM, Kafatos FC, Christophides GK: **Leucine-rich repeat protein complex activates mosquito complement in defense against *Plasmodium* parasites.** *Science* 2009, **324:**258-261.

39. Osta MA, Christophides GK, Kafatos FC: **Effects of mosquito genes on *Plasmodium* development.** *Science* 2004, **303:**2030-2032.

40. Goncalves RL, Oliveira JH, Oliveira GA, Andersen JF, Oliveira MF, Oliveira PL, Barillas-Mury C: **Mitochondrial reactive oxygen species modulate mosquito susceptibility to *Plasmodium* infection.** *PLoS ONE* 2012, **7:**e41083.

41. Meister S, Agianian B, Turlure F, Relogio A, Morlais I, Kafatos FC, Christophides GK: ***Anopheles gambiae* PGRPLC-mediated defense against bacteria modulates infections with malaria parasites.** *PLoS Pathog* 2009, **5:**e1000542.

42. Meister S, Kanzok SM, Zheng XL, Luna C, Li TR, Hoa NT, Clayton JR, White KP, Kafatos FC, Christophides GK, Zheng L: **Immune signaling pathways regulating bacterial and malaria parasite infection of the mosquito *Anopheles gambiae*.** *Proc Natl Acad Sci U S A* 2005, **102:**11420-11425.

43. Harris C, Lambrechts L, Rousset F, Abate L, Nsango SE, Fontenille D, Morlais I, Cohuet A: **Polymorphisms in *Anopheles gambiae* immune genes associated with natural resistance to *Plasmodium falciparum*.** *PLoS Pathog* 2010, **6:**e1001112.

44. Abraham EG, Pinto SB, Ghosh A, Vanlandingham DL, Budd A, Higgs S, Kafatos FC, Jacobs-Lorena M, Michel K: **An immune-responsive serpin, SRPN6, mediates mosquito defense against malaria parasites.** *Proc Natl Acad Sci U S A* 2005, **102:**16327-16332.

45. Bahia AC, Kubota MS, Tempone AJ, Araujo HR, Guedes BA, Orfano AS, Tadei WP, Rios-Velasquez CM, Han YS, Secundino NF, et al: **The JAK-STAT pathway controls *Plasmodium vivax* load in early stages of *Anopheles aquasalis* infection.** *PLoS Negl Trop Dis* 2011, **5:**e1317.

46. Molina-Cruz A, Dejong RJ, Ortega C, Haile A, Abban E, Rodrigues J, Jaramillo-Gutierrez G, Barillas-Mury C: **Some strains of *Plasmodium falciparum*, a human malaria parasite, evade the complement-like system of *Anopheles gambiae* mosquitoes.** *Proc Natl Acad Sci U S A* 2012, **109:**E1957-1962.

47. Blandin S, Shiao SH, Moita LF, Janse CJ, Waters AP, Kafatos FC, Levashina EA: **Complement-like protein TEP1 is a determinant of vectorial capacity in the malaria vector *Anopheles gambiae*.** *Cell* 2004, **116:**661-670.

48. Smith RC, Eappen AG, Radtke AJ, Jacobs-Lorena M: **Regulation of anti-*plasmodium* immunity by a LITAF-like transcription factor in the malaria vector *Anopheles gambiae*.** *PLoS Pathog* 2012, **8:**e1002965.

49. Chen Y, Dong Y, Sandiford S, Dimopoulos G: **Transcriptional mediators Kto and Skd are involved in the regulation of the IMD pathway and *anti-plasmodium* defense in *Anopheles gambiae*.** *PLoS ONE* 2012, **7:**e45580.
