# Supplementary material for: A compendium of molecules involved in vector-pathogen interactions pertaining to malaria
Source: Malar J. 2013 Jun 26;12:216. doi: 10.1186/1475-2875-12-216 (PMC3734095; doi:10.1186/1475-2875-12-216)
Supplement: Additional file 2 — Molecules that differentially affect the Plasmodium development depending on the mosquito and Plasmodium species. The file includes the list of molecules that have different effects on the malarial parasite in different mosquito and/or plasmodium species. Depending on the vector and parasite species, these molecules either aid or inhibit oocyst formation. [file 1475-2875-12-216-S2.docx]

**Additional table 2: Molecules that differentially affect the *Plasmodium* development depending on the mosquito and *Plasmodium* species**

The table includes the molecules that have different effects on the malarial parasite in different mosquito and/or *plasmodium* species. Depending on the vector and parasite species, these molecules either aid or inhibit oocyst formation

|  | **Protein** | **VectorBase ID** | **Mosquito species** | ***Plasmodium* species** | **Effect of knockdown** | **References** |
| --- | --- | --- | --- | --- | --- | --- |
| 1 | CTL4 | AGAP005335 | G3 and Yaoundé strain of *An. gambiae* | *P. falciparum* | No effect on the number of oocysts | *Cohuet, A et al., 2006.* |
|  |  |  | G3 and Yaoundé strain of *An. gambiae* | *P. berghei* | Decrease in the number of oocysts |  |
| 2 | CTLMA2 | AGAP005334 | G3 and Yaoundé strain of *An. gambiae* | *P. falciparum* | No effect on the number of oocysts | *Cohuet, A et al., 2006.* |
|  |  |  | G3 and Yaoundé strain of *An. gambiae* | *P. berghei* | Decrease in the number of oocysts |  |
| 3 | FBN6 | AGAP011231 | Keele strain of *An. gambiae* | *P. falciparum* | No effect on the number of oocysts | *Dong, Y and Dimopoulos, G, 2009.* |
|  |  |  | Keele strain of *An. gambiae* | *P. berghei* | Increase in the number of oocysts |  |
| 4 | GPRCCK1 | AGAP01022 | Yaounde´ strain of *An. gambiae* | *P. falciparum* | Decrease in the number of oocysts | *Mendes, AM et al., 2011.* |
|  |  |  | Yaounde´ strain of *An. gambiae* | ANKA strain of *P. berghei* | Increase in the number of oocysts |  |
| 5 | GSTT1 | AGAP000761 | G3 strain of *An. gambiae* | 3D7 strain of *P. falciparum* | No effect on the number of oocysts | *Jaramillo-Gutierrez, G et al., 2009.* |
|  |  |  | G3 strain of *An. gambiae* | *P. berghei* | Decrease in the number of oocysts |  |
| 6 | Hsc-3 | AGAP004192 | G3 strain of *An. gambiae* | 3D7 strain of *P. falciparum* | Decrease in the no. of oocysts | *Jaramillo-Gutierrez, G et al., 2009.* |
|  |  |  | Nijmegen  Sda500 strain of *An. stephensi* | *P. yoelii yoelii 17X* | Increase in the no. of oocysts |  |
|  |  |  | G3 strain of *An. gambiae* | *P. berghei* | Increase in the no. of oocysts | *Brandt, SM et al., 2008.* |
| 7 | LRIM1 | AGAP006348 | Nijmegen  Sda500 strain of *An. stephensi* | *P. yoelii yoelii 17X* | No effect on the number of oocysts | *Jaramillo-Gutierrez, G et al., 2009.* |
|  |  |  | G3 strain of *An. gambiae* | *P. yoelii yoelii 17X* | Increase in the number of oocysts |  |
|  |  |  | G3 and Yaoundé strain of *An. gambiae* | *P. falciparum* | No effect on the number of oocysts | *Cohuet, A et al., 2006.* |
|  |  |  | G3 and Yaoundé strain of *An. gambiae* | *P. berghei* | Increase in the number of oocysts |  |
| 8 | OXR1 | AGAP001751 | G3 strain of *An. gambiae* | 3D7 strain of *P. falciparum* | No effect on the number of oocysts | *Jaramillo-Gutierrez, G et al., 2009.* |
|  |  |  | G3 strain of *An. gambiae* | *P. berghei* | Decrease in the number of oocysts | *Jaramillo-Gutierrez, G et al., 2010.* |
| 9 | SRPN6 | AGAP009212 | G3, Yaoundé, and A69 strains of *An. gambiae* | ANKA strain of *P. berghei* | No effect on the number of oocysts | *Abraham, EG et al., 2005.* |
|  |  |  | *An. stephensi* | ANKA strain of *P. berghei* | Increase in the number of oocysts |  |
| 10 | Kto | AGAP002523 | Keele strain of *Anopheles gambiae* | NF54 strain of *P. falciparum* | Increase in the number of oocysts | *Chen, Y et al., 2012.* |
|  |  |  | Keele strain of *Anopheles gambiae* | ANKA strain of *P. berghei* | No effect on the number of oocysts |  |
| 11 | Skd | AGAP006436 | Keele strain of *Anopheles gambiae* | NF54 strain of *P. falciparum* | Increase in the number of oocysts |  |
|  |  |  | Keele strain of *Anopheles gambiae* | ANKA strain of *P. berghei* | No effect on the number of oocysts |  |

**References**

1. Cohuet A, Osta MA, Morlais I, Awono-Ambene PH, Michel K, Simard F, Christophides GK, Fontenille D, Kafatos FC: ***Anopheles* and *Plasmodium*: from laboratory models to natural systems in the field.** *EMBO Rep* 2006, **7:**1285-1289.

2. Dong Y, Dimopoulos G: ***Anopheles* fibrinogen-related proteins provide expanded pattern recognition capacity against bacteria and malaria parasites.** *J Biol Chem* 2009, **284:**9835-9844.

3. Mendes AM, Awono-Ambene PH, Nsango SE, Cohuet A, Fontenille D, Kafatos FC, Christophides GK, Morlais I, Vlachou D: **Infection intensity-dependent responses of *Anopheles gambiae* to the African malaria parasite *Plasmodium falciparum*.** *Infect Immun* 2011, **79:**4708-4715.

4. Jaramillo-Gutierrez G, Rodrigues J, Ndikuyeze G, Povelones M, Molina-Cruz A, Barillas-Mury C: **Mosquito immune responses and compatibility between *Plasmodium* parasites and *anopheline* mosquitoes.** *BMC Microbiol* 2009, **9:**154.

5. Brandt SM, Jaramillo-Gutierrez G, Kumar S, Barillas-Mury C, Schneider DS: **Use of a *Drosophila* model to identify genes regulating *Plasmodium* growth in the mosquito.** *Genetics* 2008, **180:**1671-1678.

6. Jaramillo-Gutierrez G, Molina-Cruz A, Kumar S, Barillas-Mury C: **The *Anopheles gambiae* oxidation resistance 1 (OXR1) gene regulates expression of enzymes that detoxify reactive oxygen species.** *PLoS ONE* 2010, **5:**e11168.

7. Abraham EG, Pinto SB, Ghosh A, Vanlandingham DL, Budd A, Higgs S, Kafatos FC, Jacobs-Lorena M, Michel K: **An immune-responsive serpin, SRPN6, mediates mosquito defense against malaria parasites.** *Proc Natl Acad Sci U S A* 2005, **102:**16327-16332.

8. Chen Y, Dong Y, Sandiford S, Dimopoulos G: **Transcriptional mediators Kto and Skd are involved in the regulation of the IMD pathway and anti-*plasmodium* defense in *Anopheles gambiae*.** *PLoS ONE* 2012, **7:**e45580.
