# Supplementary material for: A compendium of molecules involved in vector-pathogen interactions pertaining to malaria
Source: Malar J. 2013 Jun 26;12:216. doi: 10.1186/1475-2875-12-216 (PMC3734095; doi:10.1186/1475-2875-12-216)
Supplement: Additional file 3 — Molecules affecting Plasmodium development studied by multiple knockdowns. The file consists of molecules, which have no or negligible effect upon silencing individually. However, when these molecules are silenced in combination with some other molecules, they affect the parasitic growth either positively or negatively. [file 1475-2875-12-216-S3.docx]

**Additional table 3: Molecules affecting *Plasmodium* development studied by multiple knockdowns**

The table includes molecules which have no or negligible effect upon silencing individually. However, when these molecules are silenced in combination with some other molecules, they affect the parasitic growth either positively or negatively.

|  | **Protein** | **Protein name** | **VectorBase ID** | **References** |
| --- | --- | --- | --- | --- |
| **Oocyst number decreases upon knock-down** | | | | |
| 1 | CLIPA2 + CLIPA5 | CLIP-domain serine protease subfamily A2 and A5 double knockdown | AGAP011790 and AGAP011787 | *Volz, J et al., 2006.* |
| 2 | CTL4 + ApoII/I | C-type lectin 4 and Lipid transporter double knockdown | AGAP005335 and AGAP001826 | *Mendes, AM et al., 2008.* |
| 3 | CTL4 + CLIPA8 | C-type lectin 4 and CLIP-domain serine protease subfamily A8 double knockdown | AGAP005335 and AGAP010731 | *Volz, J et al., 2006.* |
| 4 | CTL4 + CLIPB17 | C-type lectin 4 and CLIP-domain serine protease subfamily B17 double knockdown | AGAP005335 and AGAP001648 | *Volz, J et al., 2006.* |
| 5 | CTL4 + CLIPB4 | C-type lectin 4 and CLIP-domain serine protease subfamily B4 double knockdown | AGAP005335 and AGAP003250 | *Volz, J et al., 2006.* |
| 6 | RFABG + Vg | lipophorin and vitellogenin double knockdown | AGAP001826 and AGAP004203 | *Rono, MK et al., 2010.* |
| **Oocyst number increases upon knock-down** | | | | |
| 7 | ARC P21 + P41 | Actin related 2/3 complex 21 KDa subunit P21 and Actin related 2/3 complex 41 KDa subunit P41 | AGAP001712 and AGAP008908 | *Vlachou, D et al., 2005.* |
| 8 | Cactus + APL1C | Cactus and *Anopheles* *Plasmodium*-responsive Leucine-rich repeat protein 1C double knockdown | AGAP007938 and AGAP007033 | *Riehle, MM et al., 2008.* |
| 9 | FBN8 + FBN9 + FBN39 + FBN6 | Fibrinogen domain immunolectin 8, 9, 39 and 6 multiple knockdowns | AGAP011223, AGAP011197, AGAP000806 and AGAP011231 | *Dong, Y et al., 2009.* |
| 10 | FBN9 + Caspar | Fibrinogen domain immunolectin 9 and Caspar double knockdown | AGAP011197 and AGAP006473 | *Garver, LS et al., 2009.* |
| 11 | FBN9 + FBN6 + FBN5 + FBN26 | Fibrinogen domain immunolectin 9, 6, 5 and 26 multiple knockdowns | AGAP011197, AGAP011231, AGAP011226 and AGAP012651 | *Dong, Y et al., 2009.* |
| 12 | LRIM1 + CTL4 | Leucine-Rich Immune Molecule 1 and C-type lectin 4 double knockdown | AGAP006348 and AGAP005335 | *Osta, MA et al., 2004.* |
| 13 | LRIM1 + CTLMA2 | Leucine-Rich Immune Molecule 1 and CTL mannose binding 2 double knockdown | AGAP006348 and AGAP005334 | *Osta, MA et al., 2004.* |
| 14 | LRRD7 + Caspar | Leucine-Rich Immune Molecule 2 also known as APL2 and Caspar double knockdown | AGAP005693 and AGAP006473 | *Garver, LS et al., 2009.* |
| 15 | RFABG + TEP1 | Retinoid and fatty-acid binding glycoprotein and Thioester-containing protein 1 double knockdown | AGAP001826 and AGAP010815 | *Rono, MK et al., 2010.* |
| 16 | TEP1 + Caspar | Thioester-containing protein 1 and Caspar double knockdown | AGAP010815 and AGAP006473 | *Garver, LS et al., 2009.* |
| 17 | REL1 + REL2 with partial depletion of TEP1 and LRIM1 | Relish 1 and 2 double knockdown in mosquitoes partially depleted for Thioester-containing protein 1 and Leucine-Rich Immune Molecule 1 | AGAP009515-PA and AGAP006747 | *Frolet, C et al., 2006.* |
| **Ookinete melanization increases upon knock-down** | | | | |
| 18 | CTL4 + CTLMA2 | C-type lectin 4 and CTL mannose binding 2 double knockdown | AGAP005335 and AGAP005334 | *Osta, MA et al., 2004.* |
| 19 | CLIPA2 + CLIPA5 | CLIP-domain serine protease subfamily A2 and A5 double knockdown | AGAP011790 and AGAP011787 | *Volz, J et al., 2006.* |
| 20 | CTL4 + SRPN6 | C-type lectin 4 and Serpin 6 double knockdown | AGAP005335 and AGAP009212 | *Abraham, EG et al., 2005.* |

**References**

1. Volz J, Muller HM, Zdanowicz A, Kafatos FC, Osta MA: **A genetic module regulates the melanization response of *Anopheles* to *Plasmodium*.** *Cell Microbiol* 2006, **8:**1392-1405.

2. Mendes AM, Schlegelmilch T, Cohuet A, Awono-Ambene P, De Iorio M, Fontenille D, Morlais I, Christophides GK, Kafatos FC, Vlachou D: **Conserved mosquito/parasite interactions affect development of *Plasmodium falciparum* in Africa.** *PLoS Pathog* 2008, **4:**e1000069.

3. Rono MK, Whitten MM, Oulad-Abdelghani M, Levashina EA, Marois E: **The major yolk protein vitellogenin interferes with the anti-*plasmodium* response in the malaria mosquito *Anopheles gambiae*.** *PLoS Biol* 2010, **8:**e1000434.

4. Vlachou D, Schlegelmilch T, Christophides GK, Kafatos FC: **Functional genomic analysis of midgut epithelial responses in *Anopheles* during *Plasmodium* invasion.** *Curr Biol* 2005, **15:**1185-1195.

5. Riehle MM, Xu J, Lazzaro BP, Rottschaefer SM, Coulibaly B, Sacko M, Niare O, Morlais I, Traore SF, Vernick KD: ***Anopheles gambiae* APL1 is a family of variable LRR proteins required for Rel1-mediated protection from the malaria parasite, *Plasmodium berghei*.** *PLoS ONE* 2008, **3:**e3672.

6. Dong Y, Dimopoulos G: ***Anopheles* fibrinogen-related proteins provide expanded pattern recognition capacity against bacteria and malaria parasites.** *J Biol Chem* 2009, **284:**9835-9844.

7. Garver LS, Dong Y, Dimopoulos G: **Caspar controls resistance to *Plasmodium falciparum* in diverse *anopheline* species.** *PLoS Pathog* 2009, **5:**e1000335.

8. Osta MA, Christophides GK, Kafatos FC: **Effects of mosquito genes on *Plasmodium* development.** *Science* 2004, **303:**2030-2032.

9. Frolet C, Thoma M, Blandin S, Hoffmann JA, Levashina EA: **Boosting NF-kappaB-dependent basal immunity of *Anopheles* *gambiae* aborts development of *Plasmodium berghei*.** *Immunity* 2006, **25:**677-685.

10. Abraham EG, Pinto SB, Ghosh A, Vanlandingham DL, Budd A, Higgs S, Kafatos FC, Jacobs-Lorena M, Michel K: **An immune-responsive serpin, SRPN6, mediates mosquito defense against malaria parasites.** *Proc Natl Acad Sci U S A* 2005, **102:**16327-16332.
