# Supplementary material for: A compendium of molecules involved in vector-pathogen interactions pertaining to malaria
Source: Malar J. 2013 Jun 26;12:216. doi: 10.1186/1475-2875-12-216 (PMC3734095; doi:10.1186/1475-2875-12-216)
Supplement: Additional file 4 — Molecules affecting melanization of ookinetes. The file lists the molecules, which have been shown to be involved in the process of inhibiting or promoting the parasitic growth through ookinete melanization. [file 1475-2875-12-216-S4.docx]

**Additional table 4: Molecules affecting melanization of ookinetes**

The table lists the molecules, which have been shown to be involved in the process of inhibiting or promoting the parasitic growth through ookinete melanization**.**

| **Antagonistic molecules: those that prevent the *Plasmodium* development in mosquito**  **(ookinete melanization decreases upon knock-down)** | | | | |
| --- | --- | --- | --- | --- |
|  | **Protein** | **Protein name** | **VectorBase ID** | **Reference** |
| 1 | APL1C* | *Anopheles* *Plasmodium*-responsive Leucine-rich repeat protein 1C | AGAP007033 | *Molina-Cruz, A et al., 2012.* |
| 2 | Cdc42 | Cell division cycle 42 | AGAP002440 | *Shiao, SH et al., 2006.* |
| 3 | CLIPA8* | CLIP-domain serine protease subfamily A8 | AGAP010731 | *Volz, J et al., 2006.* |
| 4 | CLIPB17* | CLIP-domain serine protease subfamily B17 | AGAP001648 | *Volz, J et al., 2006.* |
| 5 | CLIPB3* | CLIP-domain serine protease subfamily B3 | AGAP003249 | *Volz, J et al., 2006.* |
| 6 | CLIPB4* | CLIP-domain serine protease subfamily B4 | AGAP003250 | *Volz, J et al., 2006.* |
| 7 | GPRFZ2 | Frizzled-2 | AGAP010442 | *Shiao, SH et al., 2006.* |
| 8 | LRIM1* | Leucine-Rich Immune Molecule 1 | AGAP006348 | *Molina-Cruz, A et al., 2012.* |
| 9 | TEP1* | Thioester-containing protein 1 | AGAP010815 | *Blandin, S et al., 2004.*  *Molina-Cruz, A et al., 2012.* |
| ***Agonistic molecules:* those that aid *Plasmodium* development in mosquito**  **(ookinete melanization increases upon knock-down)** | | | | |
| 1 | ApoLp-III | Apolipophorin-III | AGAP013365 | *Gupta, L et al., 2010.* |
| 2 | CLIPA2* | CLIP-domain serine protease subfamily A2 | AGAP011790 | *Volz, J et al., 2006.* |
| 3 | CLIPA5* | CLIP-domain serine protease subfamily A5 | AGAP011787 | *Volz, J et al., 2006.* |
| 4 | CLSP2 | Serine protease, putative | AGAP005031 | *Shin, SW et al., 2011.* |
| 5 | CTL4 | C-type lectin 4 | AGAP005335 | *Osta, MA et al., 2004.  Cohuet A et al., 2006.* |
| 6 | CTLMA2 | CTL mannose binding 2 | AGAP005334 | *Osta, MA et al., 2004. Cohuet A et al., 2006.* |
| 7 | PGRPLC | PGN Recognition Protein LC | AGAP005203 | *Meister, S et al., 2009.* |
| 8 | SRPN6* | Serine protease inhibitor 6 (also known as serpin 6) | AGAP009212 | *Abraham, EG et al., 2005.* |
| 9 | AgaP_ AGAP004016 | - | AGAP004016 | *Pinto, SB et al., 2009.* |
| 10 | AgaP_ AGAP006914 | - | AGAP006914 | *Pinto, SB et al., 2009.* |
| 11 | AgaP_ AGAP003304 | - | AGAP003304 | *Pinto, SB et al., 2009.* |
| 12 | AgaP_ AGAP012000 | - | AGAP012000 | *Pinto, SB et al., 2009.* |
| 13 | AgaP_ AGAP001508 | - | AGAP001508 | *Pinto, SB et al., 2009.* |

*Depicts the change in rate of melanization observed in the L3-5 refractory strain of *An. gambiae*.

**References**

1. Molina-Cruz A, Dejong RJ, Ortega C, Haile A, Abban E, Rodrigues J, Jaramillo-Gutierrez G, Barillas-Mury C: **Some strains of *Plasmodium falciparum*, a human malaria parasite, evade the complement-like system of *Anopheles gambiae* mosquitoes.** *Proc Natl Acad Sci U S A* 2012, **109:**E1957-1962.

2. Shiao SH, Whitten MM, Zachary D, Hoffmann JA, Levashina EA: **Fz2 and cdc42 mediate melanization and actin polymerization but are dispensable for *Plasmodium* killing in the mosquito midgut.** *PLoS Pathog* 2006, **2:**e133.

3. Volz J, Muller HM, Zdanowicz A, Kafatos FC, Osta MA: **A genetic module regulates the melanization response of *Anopheles* to *Plasmodium*.** *Cell Microbiol* 2006, **8:**1392-1405.

4. Blandin S, Shiao SH, Moita LF, Janse CJ, Waters AP, Kafatos FC, Levashina EA: **Complement-like protein TEP1 is a determinant of vectorial capacity in the malaria vector *Anopheles gambiae*.** *Cell* 2004, **116:**661-670.

5. Gupta L, Noh JY, Jo YH, Oh SH, Kumar S, Noh MY, Lee YS, Cha SJ, Seo SJ, Kim I, et al: **Apolipophorin-III mediates antiplasmodial epithelial responses in *Anopheles gambiae* (G3) mosquitoes.** *PLoS ONE* 2010, **5:**e15410.

6. Shin SW, Zou Z, Raikhel AS: **A new factor in the *Aedes aegypti* immune response: CLSP2 modulates melanization.** *EMBO Rep* 2011, **12:**938-943.

7. Osta MA, Christophides GK, Kafatos FC: **Effects of mosquito genes on *Plasmodium* development.** *Science* 2004, **303:**2030-2032.

8. Cohuet A, Osta MA, Morlais I, Awono-Ambene PH, Michel K, Simard F, Christophides GK, Fontenille D, Kafatos FC: ***Anopheles* and *Plasmodium*: from laboratory models to natural systems in the field.** *EMBO Rep* 2006, **7:**1285-1289.

9. Meister S, Agianian B, Turlure F, Relogio A, Morlais I, Kafatos FC, Christophides GK: ***Anopheles gambiae* PGRPLC-mediated defense against bacteria modulates infections with malaria parasites.** *PLoS Pathog* 2009, **5:**e1000542.

10. Abraham EG, Pinto SB, Ghosh A, Vanlandingham DL, Budd A, Higgs S, Kafatos FC, Jacobs-Lorena M, Michel K: **An immune-responsive serpin, SRPN6, mediates mosquito defense against malaria parasites.** *Proc Natl Acad Sci U S A* 2005, **102:**16327-16332.

11. Pinto SB, Lombardo F, Koutsos AC, Waterhouse RM, McKay K, An C, Ramakrishnan C, Kafatos FC, Michel K: **Discovery of *Plasmodium* modulators by genome-wide analysis of circulating hemocytes in *Anopheles gambiae*.** *Proc Natl Acad Sci U S A* 2009, **106:**21270-21275.
