# Supplementary material for: A compendium of molecules involved in vector-pathogen interactions pertaining to malaria
Source: Malar J. 2013 Jun 26;12:216. doi: 10.1186/1475-2875-12-216 (PMC3734095; doi:10.1186/1475-2875-12-216)
Supplement: Additional file 5 — Molecules affecting sporozoite counts. The file consists of molecules that promote or inhibit the development of sporozoites. The molecules that increase the sporozoite count are agonists and those that reduce sporozoite counts are known as antagonists. [file 1475-2875-12-216-S5.docx]

**Additional table 5: Molecules affecting sporozoite counts**

The table consists of molecules that promote or inhibit the development of sporozoites. The molecules that increase the sporozoite count are agonists and those that reduce sporozoite counts are known as antagonists.

| ***Agonistic molecules*: aid the *Plasmodium* development in mosquito**  **(sporozoite count decreases upon knock-down)** | | | | |
| --- | --- | --- | --- | --- |
|  | **Protein** | **Protein name** | **VectorBase ID** | **Reference** |
| 1 | ESP | Epithelial serine protease | AGAP010240 | *Rodrigues, J et al., 2012.* |
| 2 | OXT1 | Peptide-O-xylosyltransferase 1 | AGAP005811 | *Armistead, JS et al., 2011.* |
| 3 | PRS1 | *Plasmodium* responsive salivary 1 | AGAP006102 | *Chertemps, T et al., 2010.* |
| 4 | Saglin | Saglin | AGAP000610 | *Ghosh, AK et al., 2009.* |
| 5 | RFABG | Retinoid and fatty-acid binding glycoprotein, also known as lipophorin | AGAP001826 | *Ramakrishnan, C et al., 2012.* |
| ***Antagonistic molecules*: prevent the *Plasmodium* development in mosquito**  **(sporozoite count increases upon knock-down)** | | | | |
|  | **Protein** | **Protein name** | **VectorBase ID** | **Reference** |
| 1 | SRPN6 | Serine protease inhibitor 6 (also known as serpin 6) | AGAP009212 | *Pinto, SB et al., 2008.* |
| 2 | TEP1 | Thioester-containing protein 1 | AGAP010815 | *Ramakrishnan, C et al., 2012.* |

**References**

1. Rodrigues J, Oliveira GA, Kotsyfakis M, Dixit R, Molina-Cruz A, Jochim R, Barillas-Mury C: **An epithelial serine protease, AgESP, is required for *Plasmodium* invasion in the mosquito *Anopheles gambiae*.** *PLoS ONE* 2012, **7:**e35210.

2. Armistead JS, Wilson IB, van Kuppevelt TH, Dinglasan RR: **A role for heparan sulfate proteoglycans in *Plasmodium falciparum* sporozoite invasion of *anopheline* mosquito salivary glands.** *Biochem J* 2011, **438:**475-483.

3. Chertemps T, Mitri C, Perrot S, Sautereau J, Jacques JC, Thiery I, Bourgouin C, Rosinski-Chupin I: ***Anopheles gambiae* PRS1 modulates *Plasmodium* development at both midgut and salivary gland steps.** *PLoS ONE* 2010, **5:**e11538.

4. Ghosh AK, Devenport M, Jethwaney D, Kalume DE, Pandey A, Anderson VE, Sultan AA, Kumar N, Jacobs-Lorena M: **Malaria parasite invasion of the mosquito salivary gland requires interaction between the *Plasmodium* TRAP and the *Anopheles* saglin proteins.** *PLoS Pathog* 2009, **5:**e1000265.

5. Ramakrishnan C, Rademacher A, Soichot J, Costa G, Waters AP, Janse CJ, Ramesar J, Franke-Fayard BM, Levashina EA: **Salivary gland-specific *P. berghei* reporter lines enable rapid evaluation of tissue-specific sporozoite loads in mosquitoes.** *PLoS ONE* 2012, **7:**e36376.

6. Pinto SB, Kafatos FC, Michel K: **The parasite invasion marker SRPN6 reduces sporozoite numbers in salivary glands of *Anopheles gambiae*.** *Cell Microbiol* 2008, **10:**891-898.
